# Genome Sequences of Three Colombian Helicobacter pylori Strains Isolated from Tolimense Patients

**DOI:** 10.1128/MRA.00117-20

**Published:** 2020-04-30

**Authors:** Alix A. Guevara, Roberto C. Torres, John J. Suaréz, Fabian L. Castro-Valencia, Giovanna Parra, Javier Torres, Luis Carvajal Carmona, M. Magdalena Echeverry de Polanco, Mabel E. Bohórquez

**Affiliations:** a Research Group in Cytogenetic Phylogeny and Evolution of Populations, Departments of Sciences and Health Sciences, Tolima University, Tolima, Colombia; b Infectious Diseases Research Unit, Instituto Mexicano del Seguro Social, Mexico City, Mexico; c Medicine Program, Department of Health Sciences, Tolima University, Tolima, Colombia; d Genome Center, Department of Biochemistry and Molecular Medicine, School of Medicine, University of California, Davis, California, USA; University of Maryland School of Medicine

## Abstract

We present the complete genome sequences of three Helicobacter pylori strains isolated from patients who resided in Tolima Department, Colombia, diagnosed with chronic gastritis. The genomes present an average length of 1.6 Mbp and 1,546 genes and correspond to different H. pylori subpopulations.

## ANNOUNCEMENT

Helicobacter pylori colonizes over 50% of the human population, and it is estimated that in Colombia, 70 to 80% of the adult population is infected (1). Although colonization of the gastric mucosa with H. pylori is the main known risk factor for gastric cancer, just a small percentage of infected people develop disease (2). Altered coevolution of the human host and its infecting H. pylori strain is associated with increased risk for premalignant gastric lesions (3). In Colombia, genomic studies of infecting H. pylori have shown a mixed ancestry between the European, African, and Asian origins, and some isolates diverge from the reported populations and constitute a different subgroup (4–6). We are still learning about the structure of H. pylori populations in Colombia, and isolates from more regions need to be studied. This report presents the draft genome sequences of three H. pylori strains isolated from patients with gastritis in the department of Tolima.

This study was approved by the Tolima University Bioethics Committee (act number 02 of 31 July 2018). Informed consent and histopathological diagnosis were recorded for all participants. Gastric biopsy specimens were collected from patients at Javeriana Clinic during upper gastrointestinal endoscopy as part of the treatment of dyspepsia. The gastric biopsy specimens were grown on blood agar supplemented with sodium carbonate, hydrolyzed casein, tryptone, activated carbon, 10% fresh horse blood serum, and 1% Vitox and *Campylobacter* selective supplements (Oxoid, Basingstoke, UK) at 37°C for 3 to 15 days under microaerophilic conditions. Each isolate was obtained from a single colony that was grown under the same conditions for 3 days, and genomic DNA was obtained from established growth using a DNeasy blood and tissue kit (Qiagen). Sequencing libraries were prepared with a TruSeq Nano DNA kit (Illumina), and genomes were sequenced using the 2 × 150 paired-end protocol of the Illumina NovaSeq platform (Macrogen, South Korea). Read data sets were trimmed to improve quality with the software package Trimmomatic version 0.39 (7). The genomes were assembled *de novo* with SPAdes version 3.13.1 (8) and annotated with Prokka version 1.12 (9). Ancestry of the samples was determined using fineSTRUCTURE version 4 (10) and ChromoPainter version 2 (11) based on the single nucleotide polymorphisms (SNPs) present in the core genome and using the default parameters. To calculate the population, we included as donors all those genomes included by Thorell et al. (5), Gutiérrez-Escobar et al. (4), and Muñoz-Ramírez et al. (6).

On average, the genomes have 39% GC content, 1.6 Mbp size, and 1,564 genes. Although the strains are from patients who reside in the same department, the population of each strain was different (Table 1); the GCT27 strain corresponds to a North American subpopulation with African ancestry, the strain GCT43 corresponds to a subpopulation including strains from different regions of Latin America with African ancestry, and the GCT97 strain corresponds to a Colombian subpopulation with European ancestry. These genomes provide information on the genetic population structure and the evolution of Colombian H. pylori.

**TABLE 1:** Summary of genome sequences reported

Strain	BioSample accession no.	GenBank accession no.	SRA accession no.	Diagnosis	Host origin department in Colombia	No. of contigs >0 bp	No. of contigs >1,000 bp	Genome size (bp)^*a*^	Coverage (×)	GC content (%)^*b*^	*N*_50_ (bp)^*b*^	No. of genes	Population
GCT27	SAMN13950472	CP048601	SRR11183158	Chronic active gastritis	Valle del Cauca	57	32	1,643,791	102	39.000	110,298	1,559	*hspAfrica1WAfricaNAmerica*
GCT43	SAMN13950473	CP048600	SRR11183157	Chronic active gastritis	Risaralda	68	35	1,642,398	102	39.046	98,340	1,566	*hspAfrica1SAfricaMiscAmerica*
GCT97	SAMN13950474	CP048599	SRR11183156	Chronic active gastritis	Tolima	60	40	1,656,586	103	38.847	94,297	1,569	*hspSWEuropeColombia*

a Including contigs of ≥0 bp.

b Based on contigs of ≥500 bp.

### Data availability.

The sequence read files and the genome sequences of the strains have been deposited in the GenBank database under the accession numbers shown in Table 1. These sequences represent the first described versions (CP048601.1, CP048600.1, and CP048599.1).
